
JOURNAL INFORMATION
==============================
Journal ID: Wirtschaftsdienst

Wirtschaftsdienst

EISSN: 1613-978X

ARTICLE INFORMATION
==============================
PMCID: PMC9466327
DOI: 10.1007/s10273-022-3253-x
Article ID: 3253
Article version: 1
Subjects: Zeitgespräch

Untere Einkommensgruppen noch gezielter entlasten

Provide Even More Targeted Relief for Lower Income Groups

Kritikos Alexander S. akritikos@diw.de
1
Schulze Düding Johanna jschulzedueding@diw-econ.de
2
Morales Octavio omorales@diw-econ.de
2
Priem Maximilian mpriem@diw-econ.de
2
1 grid.8465.f 0000 0001 1931 3152 DIW Berlin, Mohrenstr. 58, 10117 Berlin, Deutschland
2 DIW ECON GmbH, Mohrenstr. 58, 10117 Berlin, Deutschland
Electronic publication date: 2022 Sep 12
Print publication date: 2022
Volume: 102
Issue: 8
First page: 590
Last page: 594
Copyright: © Der/die Autor:in 2022
License: Open Access: Dieser Artikel wird unter der Creative Commons Namensnennung 4.0 International Lizenz veröffentlicht (https://creativecommons.org/licenses/by/4.0/deed.de).
Open Access wird durch die ZBW — Leibniz-Informationszentrum Wirtschaft gefördert.
License URL: https://creativecommons.org/licenses/by/4.0/

Keywords: D31, D10, D61
issue-copyright-statement: © The Author(s) 2022

==============================

Die Preise für Energie und Lebensmittel erleben aktuell außergewöhnliche Steigerungen, deren Auswirkungen für die privaten Haushalte deutlich spürbar sind. Insbesondere Haushalte mit geringen Nettoeinkommen sehen sich durch die Preisanstiege teils erheblichen Belastungen ausgesetzt. Gemäß aktuellen Inflationsprognosen würden die Preissteigerungen für die einkommensschwächsten 10 % unter Fortschreibung ihrer erwartbaren Einkommenszuwächse im Jahr 2022 eine zusätzliche Belastung von 5,3 % ihres Nettohaushaltseinkommens gegenüber dem Vorjahr bedeuten. Da die einkommensschwachen Dezile die Mehrausgaben weder durch eine Verringerung der Sparquote oder mögliche Rücklagen kompensieren noch ihr Konsumverhalten in dem entsprechenden Ausmaß verringern können, da sie primär Waren und Dienstleistungen des Grundbedarfs konsumieren, ist es essenziell, diese Haushalte zu entlasten. Die von der Bundesregierung beschlossenen Maßnahmen unterstützen bereits die privaten Haushalte, jedoch reichen sie nicht aus, um insbesondere in den einkommensschwächeren Haushalten die Mehrbelastung zu kompensieren. Weitere Hilfspakete sollten daher gezielt und effizient Haushalte in der unteren Hälfte der Einkommensverteilung entlasten.

Seit einigen Monaten befinden sich die Inflationsraten auf hohem Niveau. Beginnend mit den Lieferkettenengpässen infolge der COVID-19-Pandemie und verstärkt durch den russischen Angriffskrieg gegen die Ukraine herrscht aktuell eine außergewöhnliche Angebotsknappheit, welche die Preise steigen lässt. Der Verbraucherpreisindex des Statistischen Bundesamts, der die Preisentwicklung von Waren und Dienstleistungen misst, die von privaten Haushalten zu Konsumzwecken erworben werden, erreicht im Mai 2022 mit 7,9 % höheren Preisen gegenüber dem Vorjahresmonat den höchsten Wert im wiedervereinigten Deutschland (Statistisches Bundesamt, 2022a). Seitdem sind die Inflationsraten leicht rückläufig, verharren aber bei einem Plus von über 7 %. In aktuellen Prognosen wird von einer Jahresinflation von 7,1 % ausgegangen (Deutsche Bundesbank, 2022). Im Vergleich dazu lag die durchschnittliche Jahresinflation in den Jahren 2000 bis 2020 bei lediglich 1,4 % (Statistisches Bundesamt, 2022b).

**Octavio Morales** ist Analyst bei DIW Econ.

**Maximilian Priem** ist Manager bei DIW Econ.

## Inflation nach Konsumbereichen und haushaltsspezifischen Konsummustern

In welchem Ausmaß die Folgen der Inflation für die Haushalte in Deutschland zu spüren sind, hängt maßgeblich von den Teuerungsraten der einzelnen Konsumgüter und vom Konsumverhalten der Haushalte ab. Konsumieren Haushalte anteilig mehr von stark im Preis steigenden Gütern und Dienstleistungen, sind sie auch anteilig stärker durch die Inflation belastet.

Betrachtet man die Inflationsraten differenziert nach Konsumbereichen, ragen die Treiber der derzeitigen Entwicklung heraus. Dazu werden die Inflationsraten der 12 Konsumbereiche gemäß der internationalen Klassifikation der Verwendungszwecke des Individualverbrauchs (United Nations, 2018) sowie zusätzlich differenziert die Energiepreise betrachtet. Als Energiepreise werden zum einen die Haushaltsenergie, d. h. Strom, Gas und andere Brennstoffe, und zum anderen die Mobilitätsenergie, d. h. Kraft- und Schmierstoffe für Fahrzeuge, aufgeführt. Wie in Abbildung 1 zu erkennen, steigen vor allem die Preise für Energie und Lebensmittel. Im Mai 2022 liegen die Preise für Mobilitätsenergie 40,7 % höher als im Mai des Vorjahres, für Haushaltsenergie 36,8 % und die für Nahrungsmittel und alkoholfreie Getränke 10,7 % höher als im Mai 2021. Andere Bereiche wie Post- und Telekommunikation, Gesundheit oder Bildungswesen verzeichnen deutlich geringere Inflationsraten.

Die Konsummuster der einzelnen Haushaltsquintile, also die Haushalte entlang der Einkommensverteilung unterteilt in fünf gleichgroße Gruppen, differenziert nach denselben Konsumbereichen (vgl. Abbildung 2), machen deutlich,

Abbildung 1
### Inflationsrate im Mai 2022 nach Konsumbereichen

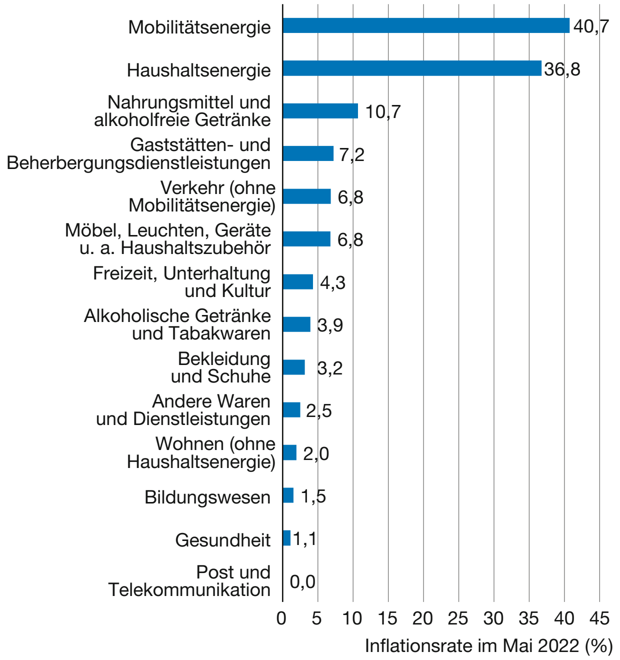

Die Inflationsrate wird gemessen als Veränderung gegenüber dem Vorjahresmonat.

dass das einkommensschwächste Quintil den Großteil seiner Konsumaufwendungen für Waren und Dienstleistungen des Grundbedarfs ausgibt.[1] Nahrungsmittel und Wohnen (inklusive Haushaltsenergie) stellen nahezu zwei Drittel des Gesamtkonsums (62,1 %) dar. Bei den einkommensstärksten 20 % der Haushalte, dem obersten Quintil, machen die Waren und Dienstleistungen des Grundbedarfs nur 44,1 % des Gesamtkonsums aus. Hinsichtlich der Ausgaben für Verkehr (inklusive Mobilitätsenergie) zeigt sich ein anderes Bild: Einkommensstarke Haushalte des obersten Quintils geben anteilig mehr Konsumausgaben für Waren und Dienstleistungen dieses Bereichs aus als das einkommensschwächste Quintil (12,7 % vs. 6,8 %). Grund dafür ist unter anderem, dass zum einen der Kauf von Fahrzeugen in diese Kategorie fällt, wobei das unterste Quintil nur selten Fahrzeuge kauft, und zum anderen, dass der Anteil an Fahrzeugeigentümer:innen im untersten Quintil deutlich geringer ist als im obersten Quintil, wodurch auch die Ausgaben für Mobilitätsenergie eine entsprechend geringere Rolle spielen.[2]

1 Zum Grundbedarf zählen Nahrung, Unterkunft und Kleidung (Christoph, 2016). Auf letztere wird in der weiteren Analyse nicht eingegangen, da die Preissteigerung in diesem Bereich relativ gering ausfällt.
2 Eigene Berechnungen auf der Grundlage des SOEP (v37) für 2020.

Abbildung 2
### Konsum nach Einkommensquintilen

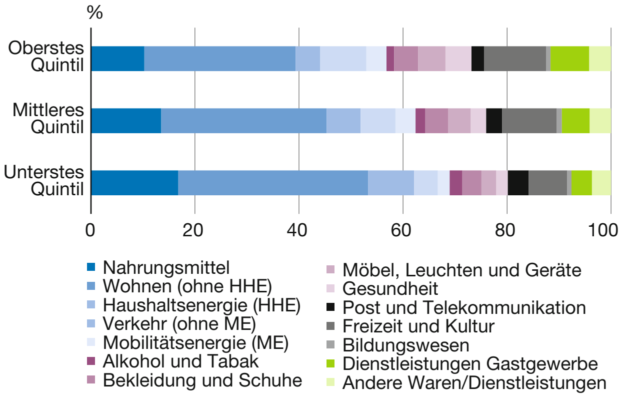

Dargestellt ist der Anteil am gesamten Konsum des jeweiligen Einkommensquintils. Gewichtet.

Quelle: eigene Berechnung auf Basis SOEPv37 und EVS 2018.

## Resultierende Belastung der Haushalte und Wirkung bestehender Entlastungsmaßnahmen

Die aus spezifischen Konsummustern resultierende Belastung durch höhere Preise für verschiedene Einkommensgruppen berechnen wir anhand eines statischen Modells auf Basis der Daten der 37. Welle des Sozio-oekonomischen Panels (SOEP) und der Einkommens- und Verbrauchsstichprobe (EVS) (Goebel et al., 2018; Forschungsdatenzentrum der Statistischen Ämter des Bundes und der Länder, 2020). Etwaige Verhaltensanpassungen der Haushalte bleiben dabei zunächst unberücksichtigt.[3] Für die Jahresinflation 2022 gehen wir von der Prognose der Bundesbank (2022) von 7,1 % aus, wonach sich angesichts der aktuellen Preissteigerungen von 7,9 % im Mai die Preisdynamik in der zweiten Jahreshälfte etwas entspannen sollte.

Die Analysen über die höheren Ausgaben unterschieden nach Einkommensdezilen im Jahr 2022 gegenüber 2021 bei einer Jahresinflation von 7,1 % zeigen, dass die Zusatzbelastung im untersten Einkommensdezil, also den einkommensschwächsten 10 %, mit 5,3 % ihres Netto-

3 Erste Berechnungen weisen darauf hin, dass es zu Verhaltensanpassungen kommt und Haushalte ihren Konsum aufgrund der gestiegenen Preise teilweise einschränken. Die Ergebnisse zeigen jedoch, dass die höchsten Belastungen von Waren und Dienstleistungen des Grundbedarfs ausgehen, die durch geringe Preiselastizitäten und damit geringe Einsparmöglichkeiten gekennzeichnet sind (Förster et al., 2021). Zudem ist bei Verhaltensanpassungen im Bereich der Haushaltsenergieversorgung mit zeitlichen Verzögerungen zu rechnen, da viele Haushalte die Abschlagszahlungen für den Energiebezug erst im Herbst erhalten (für nähere methodische Erläuterungen und tiefergehende Ergebnisse, DIW Econ, 2022).

haushaltseinkommens am größten wäre (vgl. Abbildung 3), wenn sie den gleichen Konsum wie im Vorjahr tätigen würden. Dabei werden die dezilspezifischen Wachstumsraten der Haushaltsnettoeinkommen für das Jahr fortgeschrieben. Mit 4,1 % bis 4,4 % des Nettohaushaltseinkommens ist die Belastung auch im zweiten bis vierten Einkommensdezil anhaltend hoch. Der Preisanstieg der Haushaltsenergie gefolgt von Preisanstiegen für Nahrungsmittel und Mobilitätsenergie schlägt besonders stark zu Buche.

Dieser Effekt nimmt mit steigendem Einkommen ab: Die Belastung für obere Einkommensdezile ist geringer. Die geringste Belastung erfährt das oberste Einkommensdezil mit 1,1 % seines Nettohaushaltseinkommens, beim zweitobersten Dezil liegt sie bei 3,4 %. Die Belastung der oberen Dezile wird ebenso von den Mehrausgaben für Haushaltsenergie bestimmt, jedoch folgen danach die Ausgaben für Mobilitätsenergie und dann für Nahrungsmittel. Dies ist wiederum auf die höhere Bedeutung der Verkehrsausgaben für obere Dezile gegenüber den unteren zurückzuführen.

Um die Haushalte angesichts der hohen Preissteigerungen zu entlasten, verabschiedete die Bundesregierung bereits erste Maßnahmen. In zwei Entlastungspaketen umfassen diese unter anderem die einmalige Energiepreispauschale, Einmalzahlungen für Sozialhilfeempfangende, den sogenannten Tankrabatt (Senkung der Energiesteuer auf Kraftstoffe) und die vorgezogene Abschaffung der EEG-Umlage (Ergebnis des Koalitionsausschusses, 2022a; 2022b).[4] Die Belastung der privaten Haushalte sinkt durch die Maßnahmen. Dabei ist die Wirkung der Entlastungspakete im einkommensschwächsten Dezil am höchsten: Haushalte dieses Dezils werden um 3,7 % ihres Nettohaushaltseinkommens entlastet, wodurch ihre Nettobelastung auf 1,6 % sinkt. Damit sinkt die Belastung zwar merklich, jedoch reichen die bisher beschlossenen Maßnahmen nicht aus, um die Mehrbelastung bei einer angenommenen Inflation von 7,1 % vollständig zu kompensieren. Am wirkungsvollsten für das einkommensschwächste Dezil ist die einmalige Energiepreispauschale gefolgt von Einmalzahlungen für Sozialhilfeempfangende und der Übernahme der Heizkosten.

Die Entlastungen wirken aufgrund der Steuerprogression des Entlastungsbetrags bei den einkommensschwächeren Haushalten stärker als in den anderen Dezilen.

4 Das 9-Euro-Ticket für den ÖPNV wurde in der Modellierung nicht berücksichtigt. Da andere Analysen zeigen, dass die Auswirkungen dieser Maßnahme vergleichsweise gering sind, ist davon auszugehen, dass die hier dargestellten Ergebnisse auch bei Berücksichtigung dessen bestehen bleiben würden (zur Analyse von Entlastungspaketen, Bach und Kautz, 2022).

Abbildung 3

**Be- und Entlastung durch die Inflation und staatliche Maßnahmen im Jahr 2022 nach Einkommensdezilen**

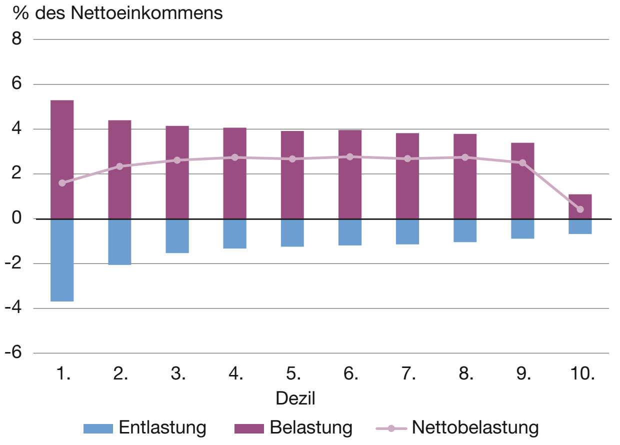

Die Belastung ist definiert als höhere Ausgaben im Jahr 2022 gegenüber 2021 in % des Nettohaushaltseinkommens. Die Entlastung ist definiert als der durch die Entlastungsmaßnahmen zusätzlich zur Verfügung stehende Anteil des Nettohaushaltseinkommens 2022 gegenüber 2021. Die Nettobelastung ergibt sich aus der Differenz dieser. Die dargestellten Größen entsprechen einer Jahresinflation von 7,1 %.

Quelle: eigene Berechnung auf Basis SOEPv37 und EVS 2018.

Dennoch fällt auf, dass die Nettobelastung mit 2,3 % des Nettohaushaltseinkommens bereits ab dem zweiten Einkommensdezil deutlich höher ist. Dies lässt vermuten, dass bereits Haushalte dieses Dezils deutlich schlechter durch die bisherigen Entlastungspakete erreicht werden. Gleichzeitig wird deutlich, dass auch die Haushalte der oberen Dezile durch die beschlossenen Maßnahmen entlastet werden. Dort ist nach der Energiepreispauschale der Tankrabatt gefolgt von der Abschaffung der EEG-Umlage am wirkungsvollsten.

## Weitere Entlastungsmaßnahmen

Sollte die Preisdynamik sich nicht wie von der Bundesbank erwartet beruhigen, ist von einer deutlich höheren Belastung auszugehen. Bereits unter der aktuellen Entwicklung bedeuten die Preissteigerungen eine teils erhebliche Mehrbelastung für die privaten Haushalte. Insbesondere vor dem Hintergrund der hohen Bedeutung der Waren und Dienstleistungen des Grundbedarfs und dementsprechend geringen Anpassungs- und Einsparmöglichkeiten verschärft sich die für viele einkommensschwache Haushalte ohnehin schon prekäre Situation deutlich. Hinzu kommt, dass Haushalte in der unteren Hälfte der Einkommensverteilung im Gegensatz zu den Haushalten in der oberen Hälfte der Einkommensverteilung die Mehrausgaben nur selten durch eine Verringerung der Sparquote oder durch Auflösung von Rücklagen auffangen können.[5]

Damit ist es geboten, dass etwaige weitere Maßnahmen zur Entlastung der privaten Haushalte zielgerichtet und effizient jene Haushalte erreichen, die auf die Unterstützung angewiesen sind. Zudem ist es notwendig, die bisherigen Maßnahmen und deren Wirkung zu evaluieren. So zeigen die Ergebnisse beispielsweise, dass der Tankrabatt vor allem einkommensstärkere Haushalte entlastet und die Wirkung in den einkommensschwächeren Dezilen eher gering ist, mithin also eine Umverteilung von unten nach oben stattfindet.

Wenn auch die anderen Maßnahmen die größte Wirkung in den einkommensschwächsten Haushalten entfalten, fallen dennoch einige Haushalte durch das Raster. Weitergehende Berechnungen zeigen, dass einkommensschwache Rentner:innen zwar eine ähnliche Belastung wie vergleichbare Haushalte von abhängig Beschäftigten erfahren, die Entlastung für diese aber deutlich geringer ist (DIW Econ, 2022). Bei neuen Maßnahmen sollte dies berücksichtigt werden.

Sollten weiter steigende Energiepreise zu zusätzlichen Belastungen für die Haushalte führen, werden derzeit verschiedene Vorgehensweisen diskutiert. Zum einen könnte eine rein marktorientierte Lösung verfolgt werden, wonach die Bundesregierung keine zusätzlichen staatlichen Unterstützungsmaßnahmen durchführt und die Energiepreise den Marktkräften überlässt, also auch z.B. den Gasversorgern die Weitergabe der Kosten gestattet. Von den dann hohen Marktpreisen würde ein erheblicher Anreiz zum Energiesparen ausgehen. Erste Schätzungen sehen hier ein Einsparpotenzial von bis zu 26% für Gas (Holz et al., 2022). Gleichzeitig müssten Haushalte, für die weitere Energieeinsparungen nicht möglich sind und die die zusätzlichen Energiemehrausgaben nicht über ihr Einkommen abfedern können, ungedeckte Kosten über die Auflösung von Vermögenspositionen finanzieren. Das ist gerade für die Geringverdienenden und die untere Mittelschicht, also Haushalte, deren Einkünfte unterhalb des Medians liegen, kaum möglich, da sie eben nicht nur geringe Verdienste haben, sondern ihnen auch kaum Vermögen zur Verfügung steht. Für manche einkommensschwache Haushalte kann dies die Zahlungsunfähigkeit und die Abkoppelung von der Energiezufuhr bedeuten, wenn sie entsprechende Nachzahlungen ihrer Energieversorger nicht leisten können. Eine Vielzahl von Privatinsolvenzen stünde ins Haus.

Dies kann in einer sozialen Marktwirtschaft nicht gewollt sein. Daher steht stattdessen als zweiter Vorschlag im Raum, Energiepreise, insbesondere den Gaspreis, zu deckeln und damit eine marktorientierte Preissetzung auszusetzen, die aktuelle Knappheiten widerspiegelt. Dieser Vorschlag hat den Vorteil einer leichten administrativen Umsetzbarkeit, ist aber mit einer Vielzahl von Nachteilen verbunden. *Erstens* stellt ein Preisdeckel eine direkte Energiesubvention dar, die für alle Haushalte die Kosten senkt, nicht nur für geringverdienende Haushalte. Sollten Haushalte in den oberen Einkommensdezilen mehr Energie verbrauchen als Haushalte in den unteren Einkommensdezilen und würde der Preisdeckel für eine relativ hohe Menge angesetzt werden,[6] würde aus dieser Subvention ähnlich dem Tankrabatt eine weitere Umverteilung von unten nach oben resultieren. *Zweitens* würde über einen solchen Preisdeckel der Anreiz zum Energiesparen verloren gehen. Die dann häufig angeführte Alternative, die Haushalte durch Appelle zum Energiesparen aufzufordern, dürfte dagegen schwächer ausfallen. In jedem Fall würde weniger Energie eingespart werden als unter der Freigabe von Energiepreisen. *Drittens* führt ein auf Gaslieferungen beschränkter Preisdeckel zu weiteren Preisverzerrungen, da dadurch die Preise für Heizöl im Verhältnis zu Gas relativ teurer würden. Geringverdienende Haushalte, die aber auf Heizöl statt Gas angewiesen sind, müssten also weiterhin anderweitige finanzielle Unterstützung bekommen. Und unter Umständen würden durch einen Gaspreisdeckel aufgrund der dann relativ niedrigen Verbauchskosten auch noch Neuinvestitionen in Gasheizungen befördert werden.

Danach wirkt ein weiterer Vorschlag als dritte Möglichkeit zielgerichteter, wonach zum einen die Energiepreise nach wie vor durch den Markt gesetzt würden, somit die Anreize zum Energiesparen erhalten blieben. Gleichzeitig sollen alle Haushalte der unteren fünf Dezile, also die Geringverdienenden und die untere Mittelschicht, die derzeit die steigenden Energiepreise weder aus ihren Einkommen noch aus ihren Vermögen vollständig decken können, durch eine weitere Energiepreispauschale entlastet werden. Mit anderen Worten: Der Erhalt der Pauschalzahlungen wird im Gegensatz zur ersten Pauschalzahlung nur bis zu einer bestimmten Höhe des Jahreseinkommens gewährt, deren Grenze sich ungefähr nach dem Medianeinkommen berechnen würde. Angesichts der Erfahrungen aus der ungleich höheren Belastung für Haushalte ab dem zweiten Einkommensdezil ist es weiterhin eine Überlegung wert, diese Einmalzahlungen nicht zu besteuern, dann würden alle Haushalte in den unteren Einkommensdezilen in gleicher Form von solchen Zahlungen profitie-

5 Eigene Berechnungen auf der Grundlage des SOEP (v37) für das Jahr 2020 (auch Deutsche Bundesbank, 2017).

6 Derzeit sind 8.000 kWh angesetzt, geringverdienende Haushalte verbrauchen pro Kopf jedoch weit weniger (Held, 2019).

ren. Um einen harten Übergang zwischen fünften und sechstem Dezil zu vermeiden, ist für das sechste Dezil die Gewährung einer dann mit zusätzlichen Einkünften rasch sinkenden Pauschalzahlung überlegenswert. Haushalte mit geringen Einkommen, die derzeit nicht als abhängig Beschäftigte oder Selbständige über die Steuerbehörden erreicht werden können, müssen dann noch über die Gewährung einer Heizkostenpauschale in gleicher Höhe unterstützt werden. Insgesamt wäre diese staatliche Intervention, von der nur die untere Hälfte der Einkommensverteilung profitieren würde, wohl kostengünstiger als eine direkte Energiepreissubvention, von der alle Haushalte profitierten. Gleichzeitig bliebe der Anreiz zum Energiesparen erhalten und würde eine hohe Zahl von privaten Insolvenzen in den unteren Dezilen der Einkommensverteilung vermieden. Auch Fehlanreize für Investitionen in Richtung subventionierter Energieträger würden vermieden. Appelle zum Energiesparen können auch dann eine nützliche Ergänzung sein.

**Title:** ***Provide Even More Targeted Relief for Lower Income Groups***

Prices for energy and food are currently rising extraordinarily sharply. Households with low net incomes in particular are being burdened by the price increases, in some cases dramatically. According to current inflation forecasts, price increases for the lowest-income ten percent would mean a burden of 5.3 percent of their net household income in 2022. Since the low-income deciles cannot compensate for the additional expenditure by reducing their savings rate or possible reserves, nor can they restrict their consumption behavior to a corresponding extent since they primarily consume goods and services for basic needs, it is essential to provide relief for these households. The measures already adopted by the German government are easing the burden on citizens, but they are not enough to compensate for the additional burden on lower-income households. Further aid packages should provide targeted and efficient relief for households at the lower half of the income distribution.

Prof. Dr. Alexander S. Kritikos ist Forschungsdirektor am DIW Berlin und Professor für Volkswirtschaftslehre an der Universität Potsdam.

Johanna Schulze Düding ist Analystin bei DIW Econ.

Octavio Morales ist Analyst bei DIW Econ.

Maximilian Priem ist Manager bei DIW Econ.


Literatur

Bach S Knautz J Hohe Energiepreise: Ärmere Haushalte werden trotz Entlastungspaketen stärker belastet als reichere Haushalte DIW Wochenbericht 2022 17 243 252
Christoph, B. (2016), Materielle Lebensbedingungen im Grundsicherungsbezug, WSI Mitteilungen, 344–352, https://www.wsi.de/data/wsimit_2016_05_christoph.pdf (29. Juni 2022).
Deutsche Bundesbank (2017), Die Studie zur wirtschaftlichen Lage privater Haushalte (PHF), https://www.bundesbank.de/de/bundesbank/forschung/haushaltsstudie/studie-zur-wirtschaftlichen-lage-privaterhaushalte-604850 (8. August 2022).
Deutsche Bundesbank (2022), Perspektiven der deutschen Wirtschaft für die Jahre 2022 bis 2024, Monatsbericht, Juni, 15–47, https://www.bundesbank.de/de/presse/pressenotizen/bundesbank-projektionen-wirtschaftserholung-duerfte-sich-fortsetzen-892554 (8. August 2022).
DIW Econ (2022), Belastung einkommensschwacher Haushalte durch steigende Inflation, https://diw-econ.de/wp-content/uploads/KEx_Diakonie_DIWEcon_v4.0.pdf (8. August 2022).
Ergebnis des Koalitionsausschusses (2022a), 10 Entlastungsschritte für unser Land, 23. Februar, https://www.bundesflnanzministerium.de/Content/DE/Downloads/Oeffentliche-Finanzen/10-entlastungsschritte-fuer-unser-land.pdf?__blob=publicationFile&v=4 (29. Juni 2022).
Ergebnis des Koalitionsausschusses (2022b). Maßnahmenpaket des Bundes zum Umgang mit den hohen Energiekosten, 23. März, https://www.bundesfinanzministerium.de/Content/DE/Downloads/2022-03-23-massnahmenpaket-bund-hohe-energiekosten.pdf?__blob=publicationFile&v=6 (8. August 2022).
Forschungsdatenzentrum der Statistischen Ämter des Bundes und der Länder (2020), DOI: 10.21242/63221.2018.00.08.3.1.0.
Förster, H., K. Hünecke, V. Liste und K. Schumacher (2021), Auswirkungen des Klimawandels im Bereich Ernährung — Verteilungswirkungen am Beispiel von Nahrungsmittelgruppen, Forschungsbericht, Bundesministerium für Arbeit und Soziales, FB583, Öko-Institut e. V. https://www.ssoar.info/ssoar/handle/document/75718 (29. Juni 2022).
Goebel, J., M. M. Grabka, S. Liebig, M. Kroh, D. Richter, C. Schröder, und J. Schupp (2018), The German Socio-Economic Panel (SOEP), Journal of Economics and Statistics, 345–260, 10.1515/jbnst-2018-0022.
Held, B. (2019), Einkommensspeziflsche Energieverbräuche privater Haushalte. Eine Berechnung auf Basis der Einkommens-und Verbrauchstichprobe, WISTA Wirtschaft und Statistik.
Holz, F., R. Sogalla, C. Kemfert und C. von Hirschhausen (2022), Energieversorgung in Deutschland auch ohne Erdgas aus Russland gesichert, DIW aktuell, 83, https://www.diw.de/de/diw_01.c838843.de/publikationen/diw_aktuell/2022_0083/energieversorgung_in_deutschland_auch_ohne_erdgas_aus_russland_gesichert.html (8. August 2022).
Statistisches Bundesamt (2022a), Verbraucherpreisindizes für Deutschland, Monatsbericht, Mai 2022, Fachserie 17, Reihe 7, https://www.destatis.de/DE/Themen/Wirtschaft/Preise/Verbraucherpreisindex/Publikationen/Downloads-Verbraucherpreise/verbraucherpreisem-2170700221054.pdf?__blob=publicationFile (8. August 2022).
Statistisches Bundesamt (2022b), Verbraucherpreisindizes für Deutschland, Jahresbericht, 2021, https://www.destatis.de/DE/Themen/Wirtschaft/Preise/Verbraucherpreisindex/Publikationen/Downloads-Verbraucherpreise/verbraucherpreisindex-jahresbericht-pdf-5611104.pdf?__blob=publicationFile (8. August 2022).
United Nations (2018), Classification of Individual Consumption According to Purpose (COICOP), Statistical Papers Series M, 99, https://unstats.un.org/unsd/classifications/unsdclassifications/COI-COP_2018_-_pre-edited_white_cover_version_-_2018-12-26.pdf (29. Juni 2022).
